# Economic evaluation of the introduction of the Prostate Health Index as a rule-out test to avoid unnecessary biopsies in men with prostate specific antigen levels of 4-10 in Hong Kong

**DOI:** 10.1371/journal.pone.0215279

**Published:** 2019-04-16

**Authors:** Janet Bouttell, Jeremy Teoh, Peter K. Chiu, Kevin S. Chan, Chi-Fai Ng, Robert Heggie, Neil Hawkins

**Affiliations:** 1 Health Economics and Health Technology Assessment, Institute of Health and Wellbeing, University of Glasgow, Glasgow, United Kingdom; 2 S.H. Ho Urology Centre, Department of Surgery, Prince of Wales Hospital, The Chinese University of Hong Kong, Hong Kong SAR, China; Carolina Urologic Research Center, UNITED STATES

## Abstract

A recent study showed that the Prostate Health Index may avoid unnecessary biopsies in men with prostate specific antigen 4-10ng/ml and normal digital rectal examination in the diagnosis of prostate cancer in Hong Kong. This study aimed to conduct an economic evaluation of the impact of adopting this commercially-available test in the Hong Kong public health service to determine whether further research is justified. A cost-consequence analysis was undertaken comparing the current diagnostic pathway with a proposed diagnostic pathway using the Prostate Health Index. Data for the model was taken from a prospective cohort study recruited at a single-institution and micro-costing studies. Using a cut off PHI score of 35 to avoid biopsy would cost HK$3,000 and save HK$7,988 per patient in biopsy costs and HK$511 from a reduction in biopsy-related adverse events. The net cost impact of the change was estimated to be HK$5,500 under base case assumptions. At the base case sensitivity and specificity for all grades of cancer (61.3% and 77.5% respectively) all grade cancer could be missed in 4.22% of the population and high grade cancer in 0.53%. The introduction of the prostate health index into the diagnostic pathway for prostate cancer in Hong Kong has the potential to reduce biopsies, biopsy costs and biopsy-related adverse events. Policy makers should consider the clinical and economic impact of this proposal.

## Introduction

Prostate Cancer (PCa) is the second most commonly diagnosed cancer in men worldwide [1]. The incidence of PCa in Chinese men is 10 times lower than the rate in men from Western Europe but it has increased rapidly in recent years [2,3]. Positive biopsy rates are lower in Asian men (15–25%) compared with Western European men (30%) and cancer tends to be diagnosed later [2,3]. The first steps on the current diagnostic pathway in Hong Kong to evaluate for PCa is a digital rectal examination (DRE) and the prostate specific antigen (PSA) blood test. In men whose DRE is normal but whose PSA levels are between 4–10 ng/ml the current diagnostic pathway requires a transrectal ultrasound-guided (TRUS) biopsy. Such biopsies are invasive and carry considerable risks of post-procedure complications including infection, fever, acute urinary retention, haematuria and haemospermia. As positive biopsy rates are low, many biopsies are carried out unnecessarily under the current diagnostic set-up [2].

The Prostate Health Index (PHI) is a commercially available blood test manufactured by Beckman Coulter Inc. approved by the United States Food and Drug Administration (FDA) in 2012 for use in patients with PSA of 4–10 ng/mL and normal DRE. The test uses a combination of different forms of PSA (tPSA, fPSA and [–2] proPSA(p2PSA) and has shown improved ability to predict presence of PCa and clinically significant PCa at biopsy compared to total PSA [4,5].

Given the improved performance of PHI over total PSA, it has been proposed as a rule-out test prior to prostate biopsy. The purpose of this study is to provide a preliminary indication of economic impact were the PHI test to be integrated into the diagnostic pathway prior to prostate biopsy for men in Hong Kong.

## Materials and methods

### Diagnostic pathway

**Fig 1:**
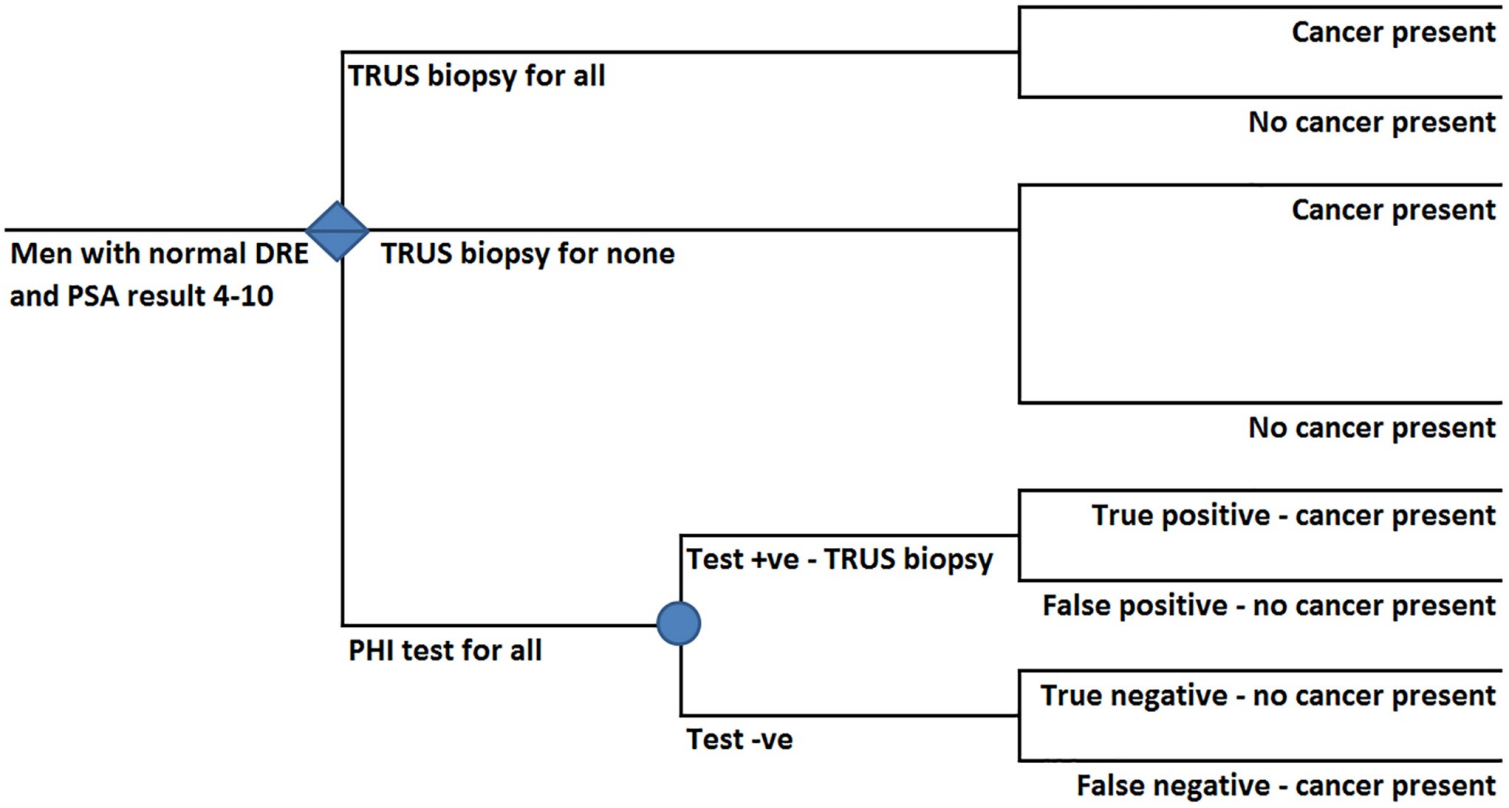
Decision tree illustrating diagnostic strategies for men with suspected prostate cancer, normal digital rectal examination and prostate specific antigen level 4–10 in Hong Kong. DRE–Digital Rectal Examination, PHI–Prostate Health Index, PSA—Prostate Specific Antigen, TRUS–Transrectal Ultrasound-guided biopsy. Diamond represents a decision node. Circle represents a probability node.

We mapped the current strategy (biopsy all) and the proposed diagnostic strategy for men with normal DRE and PSA levels 4–10 ng/ml using information supplied by Hong Kong clinicians (JT, PC and CFN). For the proposed strategy, we estimated the costs and consequences of three different cut-off levels for the test. These pathways were set out as decision trees with a time horizon covering the diagnostic process up to biopsy (see Fig 1). A Hong Kong public health service perspective was adopted. Health outcomes differ between the strategies due to the direct impact of the biopsy and adverse effects following a proportion of biopsies and the proportion of cancer cases that are missed. Health outcomes relating to the biopsy procedure itself or complications of the procedure are not considered in this study but they would be positive under each of the testing strategies as they reduce as the number of biopsies reduces. We calculate and present the proportion of missed cancers which would likely result from each testing strategy as well as the costs of the alternative strategies.

Fig 1 shows the current strategy under which all patients undergo TRUS biopsy and the proposed testing strategy. In the proposed pathway (the lowest branch of the decision tree) all patients are tested using PHI. Those with a positive result undergo TRUS biopsy. Those with PHI scores under the threshold (negative result) do not undergo biopsy. In both positive and negative test result arms there is the possibility of the test result being correct or incorrect resulting in missed cases of cancer as well as a proportion of unnecessary biopsies.

### Data sources

**Fig 2:**
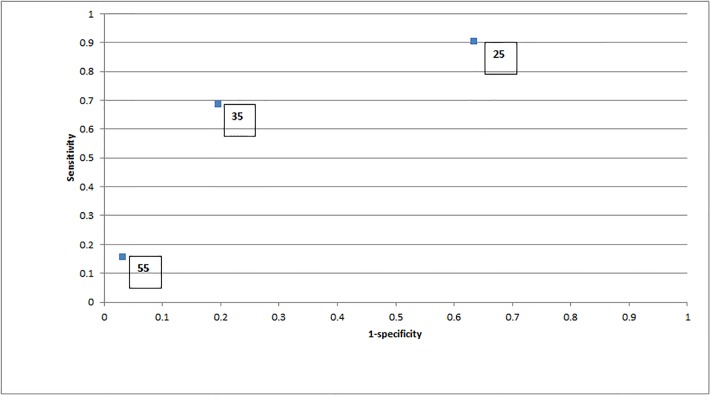
Test performance of Prostate Health Index at cut-off levels of 25, 35 and 55. Figures in boxes are the cut-off levels for the Prostate Health Index test from Chiu et al study [2]. Sensitivity/specificity for any grade cancer at different cut-off levels are 88.7%/36.5% at 25, 61.3%/77.5% at 35 and 12.9%/97.4% at 55.

Clinical data to populate the decision trees and costing data for all costs apart from the PHI test were collected as part of the study reported by Chiu and colleagues [2] (see Table 1 for inputs to the model). Sensitivity and specificity for three different thresholds of PHI score are shown in Fig 2. Any accident and emergency attendances and length of hospital stay (where appropriate) following biopsy were recorded. This resource usage was valued using costs from the Annual Report for 2016–7 of the Hospital Authority [6]. The PHI test cost was based on the cost of the test at a Hong Kong clinic to a private user. Costs are not discounted given the short time horizon of the economic evaluation. All data were fully anonymized before access. The ethics committee waived the requirement for informed consent in this study.

**Table 1:** Inputs to the model and data sources.

**Clinical data from Chiu et al study** [2]	
Cohort size–men recruited between April 2008 and July 2015 with PSA 4-10ng/mL and negative DRE—undergoing biopsy	569
Prevalence of high-grade cancer in men with PHI score <25 (1/192)	0.5%
Prevalence of any grade cancer in men with PHI score <25 (7/192)	3.6%
Prevalence of high-grade cancer in men with PHI score 25–35 (2/225)	0.9%
Prevalence of any grade cancer in men with PHI score 25–35 (17/225)	7.6%
Prevalence of high-grade cancer in men with PHI score 35–55 (9/131)	6.9%
Prevalence of any grade cancer in men with PHI score 35–55 (30/131)	22.9%
Prevalence of high-grade cancer in men with PHI score >55 (4/21)	19.0%
Prevalence of any grade cancer in men with PHI score >55 (8/21)	38.1%
Prevalence of high-grade cancer in full cohort (16/569)	2.8%
Prevalence of any grade cancer in full cohort (62/569)	10.9%
**Clinical data from retrospective analysis of Chiu et al cohort (unpublished)**	
Proportion of patients attending Accident and Emergency after biopsy (39 patients from 569 undergoing biopsy)	0.07
Proportion of patients hospitalised after Accident and Emergency attendance (15 patients from 569 undergoing biopsy)	0.38
Mean length of stay in hospital following adverse event after biopsy	4.67 days
**Costs**	**HK$**
Cost of PHI test to private patient at a Hong Kong clinic (unpublished)	3,000
Cost of Accident and Emergency Department Attendance [6]	1,300
Cost of hospitalisation following biopsy (4.67 days at HK$4,950 per day) [6]	23,116
Cost of TRUS biopsy from hospital finance department analysis (unpublished)	10,900

DRE–Digital Rectal Examination, PHI–Prostate Health Index, TRUS–Transrectal Ultrasound-guided

### Sensitivity and threshold analysis

One-way sensitivity analysis was undertaken whereby input parameters were varied in turn by 50% of base case value in both directions (whilst all other inputs to the model were held constant) to determine the impact of an over or under-estimation on the base-case results. This form of sensitivity analysis was undertaken as this allowed decision makers to assess the individual impact of each of the input parameters. Where sensitivity analysis showed that the result was sensitive to an individual parameter threshold analysis was undertaken. This involved varying the range to determine the highest level at which the cost savings would be reduced to zero.

## Results

Our base-case analysis uses a PHI score of 35 as this was considered the most likely cut-off level to be acceptable to decision-makers. The introduction of the PHI test at a cut-off of 35 into the diagnostic pathway for men with normal DRE and PSA levels 4–10 ng/ml would save an estimated HK$5,500 per patient (see Table 2). The majority of the saving results from approximately 75% of patients avoiding TRUS biopsy as their PHI score was under 35. A further cost saving of HK$511 per patient results from a reduction in adverse events following biopsies. If the cut-off for PHI testing was increased to 55 over 95% of biopsies could be avoided resulting in an overall cost saving estimated to be in excess of HK$8,000 per patient. At this cut-off level cost savings are reduced to HK$914. At all cut-off levels the introduction of PHI results in cost savings although these are greater at higher cut-off levels as more biopsies are avoided. Testing costs for PHI are included in the analysis at HK$3,000 and it is proposed to test all patients in this population. Testing costs may reduce under the new testing strategy as a result of increased volumes. This has not been reflected in the base-case analysis although test cost is varied in the sensitivity and threshold analyses.

**Table 2:** Cost-consequence analysis of alternative diagnostic strategies.

Strategy	Biopsy rate	Cost of biopsies (HK$)	Cost of PHI test (HK$)	Cost of adverse events (HK$)	Total cost (HK$)	Cost savings compared to biopsy all	Missed cancer cases—all	Missed cancer cases—high grade Gleason 7 or above
Biopsy all (current strategy)	100%	10,900	0	698	11,598	-	-	-
PHI test for all—cut off 25	66.26%	7,222	3,000	463	10,685	-914	1.23%	0.18%
PHI test for all—cut off 35	26.71%	2,912	3,000	187	6,098	-5,500	4.22%	0.53%
PHI test for all—cut off 55	3.69%	402	3,000	26	3,428	-8,170	9.49%	2.11%

PHI–Prostate Health Index, HK$—Hong Kong dollars

**Fig 3:**
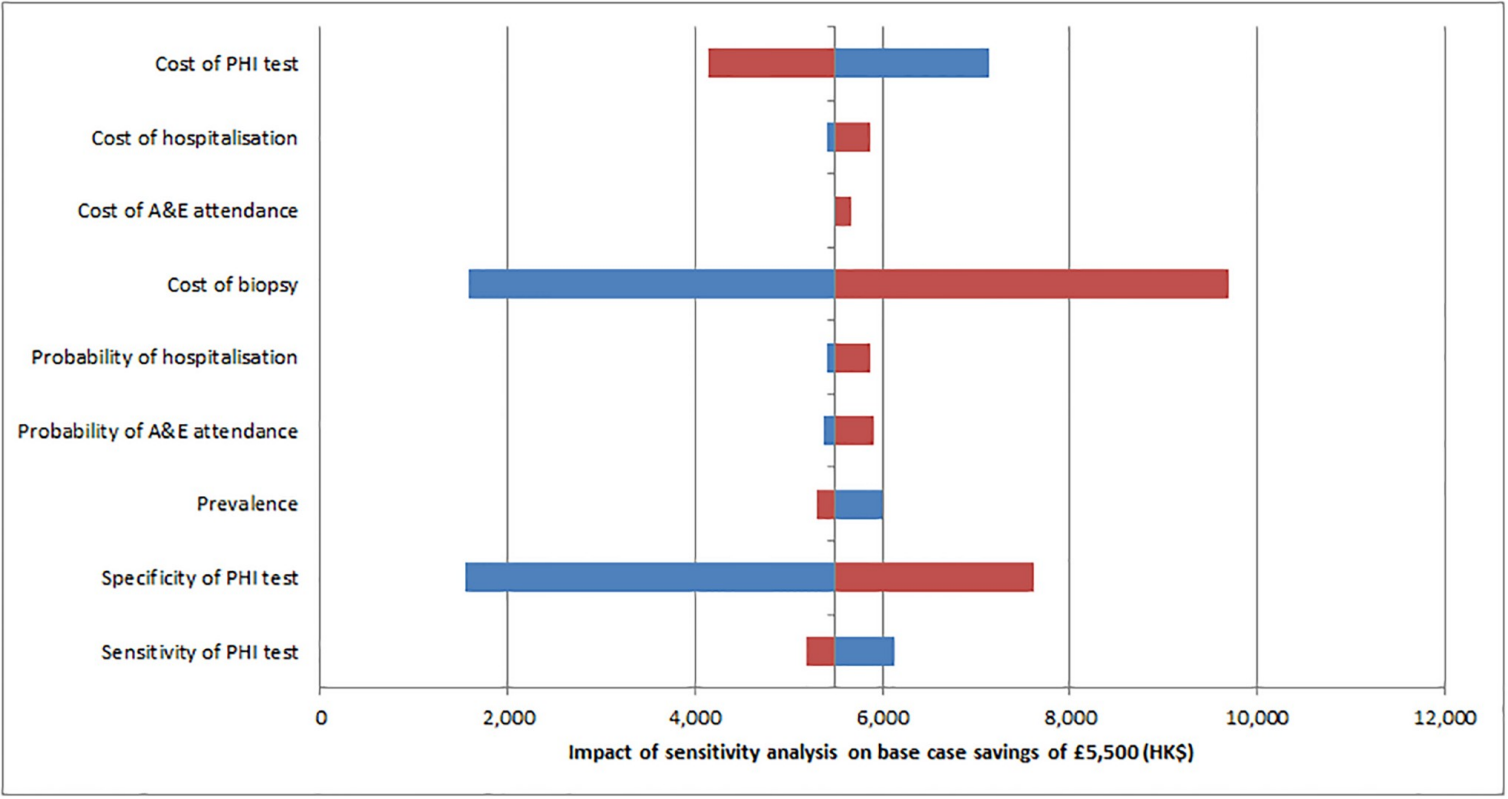
Impact of sensitivity analysis. Each parameter plus/minus 50% PHI–Prostate Health Index, A&E–accident and emergency department, HK$—Hong Kong dollars. Blue reflects the lower bound sensitivity and red the upper bound. Vertical axis crosses at base case savings of HK$5,500.

Sensitivity analysis showed that the overall cost saving was sensitive to the cost of biopsy, the cost of the PHI test and the specificity of the PHI test (see Fig 3). No individual parameter, when varied within a range 50% above or below the base case would alter the conclusion that the introduction of the test is likely to be cost saving. Threshold analysis for these parameters determined values at which the proposed strategy would be cost neutral. These were 24% for the specificity of the PHI test (base case 77.5%), HK$3,400 for cost of biopsy (base case HK$10,900) and HK$8,500 for the cost of the test (base case HK$3,000). Table 3 summarises the base case results, Table 4 the results of sensitivity analysis and Table 5 results of the threshold analyses. Reducing the sensitivity of the PHI test whilst holding specificity constant results in more missed cases (all grades of cancer in 7.2% of the population missed at 34% sensitivity, compared to 4.2% in the base case of 61%) but increases cost savings as more biopsies are avoided. Lower specificity (with constant sensitivity) results in the same level of missed cases but savings are reduced as less biopsies are avoided.

**Table 3:** Base-case results (PHI cut-off 35).

Base case	
Cancer cases missed–all grades(high grade)	**n = 24/569 (3/569) or 4.2% (0.5%)**
Cost saving per patient	**HK$****5,500**
*Made up of*:	
Additional costs of testing	(**HK$**3,000)
Direct cost savings from biopsies (circa 73% of patients at HK$10,900)	**HK$**7,988
Cost savings from reduction in adverse events	**HK$**511

HK$—Hong Kong Dollars, PHI–Prostate Health Index

**Table 4:** Sensitivity analysis.

Sensitivity analysis	Base case	Range (+/- 50%)		Results of sensitivity analysis (HK$)	
		Lower	Upper	Lower	Upper
Sensitivity of PHI test	61%	31%	100%	5,888	5,011
Specificity of PHI test	78%	39%	100%	1,499	7,824
Prevalence of all grades of cancer	11%	5%	16%	5,748	5,257
Proportion of patients experiencing adverse events	7%	3%	10%	5,242	5,757
Proportion of patients with adverse event requiring hospital	38%	19%	58%	4,831	5,724
Proposed test costs (HK$)	3,000	1,500	4,500	7,000	4,000
Cost of biopsy (HK$)	10,900	5,450	16,350	1,506	9,494
Costs of adverse events without hospitalisation (HK$)	1,300	650	1,950	5,467	5,533
Costs of adverse events with hospitalisation (HK$)	23,116	11,558	34,674	5,277	5,723

HK$—Hong Kong Dollars, PHI–Prostate Health Index

**Table 5:** Threshold analysis.

Threshold analysis	Base case	Value for proposed strategy to be cost neutral
Specificity of PHI test (at 61% sensitivity)	78%	24%
Proposed test costs (HK$)	3,000	8,500
Cost of biopsy (HK$)	10,900	3,400

HK$—Hong Kong Dollars, PHI–Prostate Health Index

## Discussion

### Summary of findings

This study found that the adoption of the PHI test for patients with negative DRE and PSA score 4–10 ng/ml in Hong Kong has the potential to deliver significant cost savings although there are implications with a proportion of all grade cancers missed. The cost savings arise because the PHI test stratifies men into those requiring TRUS biopsy and those who can avoid biopsy and enter the monitoring programme. As biopsy and the adverse events associated with it are expensive to deal with, a strategy which avoids a relatively small proportion of biopsies has the potential to deliver savings which exceed the costs of testing all the patients in this population but this must be balanced with the risks of missed cases and the longer term cost and clinical outcomes. The cost savings are sensitive to the cost of the test and the biopsy as well as the specificity of the PHI test in this population. This information is useful to decision-makers as the costs of the biopsy are known, the cost of the test would be known prior to introduction and cost savings are likely even at relatively low level of specificity (break-even level at 24% specificity compared to the base case of 78%). This information allows future evidence generation and decision-making to focus on the consequences of introducing PHI rather than the costs.

At the base case cut-off of 35, the study data [2] indicated that cancer in 4.2% of the population (including 0.53% with high grade cancers) may be missed. However, applying a cut-off of 55 to study data, prostate cancer in 9.5% of the population would have been missed including 2.1% with high grade cancer. If the cut-off is reduced to 25 around a third of biopsies could be avoided with just over 1% of all cancer cases missed including less than 0.2% of high-grade cancers.

### How these results compare to previous studies

The costs results are broadly consistent with three previous economic evaluations of PHI. The first study by Nichol et al [7] was a budget impact analysis of PHI plus total PSA and percent free PSA compared to PSA alone. This study evaluated the impact on 1 year total costs of PHI plus PSA to PSA alone in a screening programme from a US societal perspective in men 50–75 years old. Using thresholds for PHI testing of 2ng/ml and 4ng/ml they estimated cost savings of $356,647 and $94,219 respectively in a notional insurance company cohort of 100,000 men. 90% of the overall savings came from avoiding unnecessary biopsies. A further study by Nichol et al [8] extended their previous analysis to a cost-utility analysis with a 25-year time horizon. This extended analysis found that PHI plus PSA dominated PSA alone strategy for both 2ng/ml and 4ng/ml thresholds delivering cost savings of $1,199 and $443 respectively together with utility gains of 0.08 and 0.03. Both Nicholl et al studies used data relevant to the US population and are not directly applicable to a Chinese population. The final economic evaluation study identified was Heijnsdijk et al [9] who assessed the cost-effectiveness of using a PHI cut-off of 25 as an add-on to PSA with a cut-off of 3ng/ml in a European screening population aged 50–75. This study found a reduction in negative biopsies of 23%, a reduction of 17% in costs of diagnosis and 1% in total cost of prostate cancer.

A recent study [10] extended the Hong Kong cohort from Chui et al [2] with cohorts from Taiwan, Singapore and Shanghai to make a total of 1,150 men. This later study found higher prevalence of all PCa (13.1% compared to 10.9% in this study) and high grade PCa (5.7% compared to 2.8% in this study). Introduction of a test such as PHI into a population where there was a higher prevalence would mean that less biopsies could be avoided for the same risk of false negatives.

Nicholl at al [7] suggests that the negative consequences of missed cases are limited as they are likely to be found in subsequent screenings [11]. Effectively false negatives represent delayed rather than missed diagnoses. Moreover, cancers missed tend to have relatively low Gleason scores and most cancers found 2–4 years after an initial screen are still curable [12–18]. It is proposed that men below the PHI score of 35 would undergo an annual PSA test until the age of 78 when mean survival from PCa exceeds life expectancy [19,20]. However, the extent and grade of false negatives would require further study.

### Strengths of this study

This study is a preliminary cost-consequence analysis that indicates that the PHI test is potentially cost-effective in that it is not “dominated” by current practice (i.e. costs more with worse outcomes) [21]. This represents a necessary but not sufficient condition for cost-effectiveness. As far as we are aware, it is the first study to examine the potential economic impact of introducing the PHI test in an Asian population.

The study demonstrates the use of simple economic evaluation in a preliminary assessment of a diagnostic technology using local data. Another strength of the study is that it was relatively quick and resource light as a result of the simplicity of the model and the availability of locally relevant data from a previous study. Evidence was taken from a single clinical study [2] and micro-costings from a single hospital. One-way sensitivity analysis was appropriate as it allowed decision makers to assess the importance of individual parameters.

### Limitations

A significant limitation of this study is that data have been taken from a retrospective analysis of a cohort taken from a single clinical study [2].

A simplifying assumption was made that all men in the population currently undergo TRUS biopsy. In a proportion of cases patients and clinicians decide that biopsy is not their preferred option. In order to change the conclusion of our base case analysis (at a PHI cut-off of 35) just under 50% of men would need to refuse biopsy. We believe the proportion of men not undergoing biopsy is substantially lower than this.

## Conclusions

The immediate implication of this study for policy-makers is that in the Hong Kong context PHI is likely to be a cost-saving addition to the diagnostic set-up for prostate cancer in men with PSA levels of 4-10ng/ml and negative DRE. Although health outcomes have not been fully quantified, the analysis suggests that, at the proposed cut off of 35, sensitivity could be retained such that all grade cancer would be missed in 4.2% of the population (and high grade cancer in 0.53%) whilst a high proportion of biopsies would be avoided. The use of the PHI test in Hong Kong appears to warrant further investigation, particularly with regard to the level of missed cases and the longer term health outcomes in these cases. Policy makers in other jurisdictions may also wish to evaluate the use of Prostate Health Index in the diagnosis of prostate cancer particularly in Asian populations where the prevalence of prostate cancer is relatively low [10].
